# Lung Inflammation, Injury, and Proliferative Response after Repetitive Particulate Hexavalent Chromium Exposure

**DOI:** 10.1289/ehp.0900715

**Published:** 2009-08-19

**Authors:** Laura M. Beaver, Erik J. Stemmy, Arnold M. Schwartz, Jesse M. Damsker, Stephanie L. Constant, Susan M. Ceryak, Steven R. Patierno

**Affiliations:** 1 Department of Pharmacology and Physiology; 2 Institute of Biomedical Sciences; 3 Department of Microbiology, Immunology, and Tropical Medicine; 4 Department of Pathology; 5 Department of Medicine and; 6 GW Cancer Institute, George Washington University Medical Center, Washington, DC, USA

**Keywords:** chromium, hexavalent, inflammation, injury, intranasal, lung, proliferation, repair

## Abstract

**Background:**

Chronic inflammation is implicated in the development of several human cancers, including lung cancer. Certain particulate hexavalent chromium [Cr(VI)] compounds are well-documented human respiratory carcinogens that release genotoxic soluble chromate and are associated with fibrosis, fibrosarcomas, adenocarcinomas, and squamous cell carcinomas of the lung. Despite this, little is known about the pathologic injury and immune responses after repetitive exposure to particulate chromates.

**Objectives:**

In this study we investigated the lung injury, inflammation, proliferation, and survival signaling responses after repetitive exposure to particulate chromate.

**Methods:**

BALB/c mice were repetitively treated with particulate basic zinc chromate or saline using an intranasal exposure regimen. We assessed lungs for Cr(VI)-induced changes by bronchoalveolar lavage, histologic examination, and immunohistochemistry.

**Results:**

Single exposure to Cr(VI) resulted in inflammation of lung tissue that persists for up to 21 days. Repetitive Cr(VI) exposure induced a neutrophilic inflammatory airway response 24 hr after each treatment. Neutrophils were subsequently replaced by increasing numbers of macrophages by 5 days after treatment. Repetitive Cr(VI) exposure induced chronic peribronchial inflammation with alveolar and interstitial pneumonitis dominated by lymphocytes and macrophages. Moreover, chronic toxic mucosal injury was observed and accompanied by increased airway pro-matrix metalloprotease-9. Injury and inflammation correlated with airways becoming immunoreactive for phosphorylation of the survival signaling protein Akt and the proliferation marker Ki-67. We observed a reactive proliferative response in epithelial cells lining airways of chromate-exposed animals.

**Conclusions:**

These data illustrate that repetitive exposure to particulate chromate induces chronic injury and an inflammatory microenvironment that may promote Cr(VI) carcinogenesis.

Lung cancer is the leading cause of cancer deaths in both men and women in the United States (American Cancer Society 2008). Worldwide, it has been estimated that 20–30% of males and 5–20% of females of working age have been occupationally exposed to agents that cause lung cancer (World Health Organization 2002). Chronic inflammation has been implicated in the development of a wide array of human cancers, including lung cancer (reviewed by Coussens and Werb 2002). Inflammatory cells and their chemical mediators are key participants in the generation of a tumor microenvironment that promotes angiogenesis and participates in cancer metastases (Albini and Sporn 2007). Individuals with underlying inflammation in the lung due to chronic infection, asbestos exposure, or development of interstitial lung disease and asthma are at an increased risk for lung cancer (Santillan et al. 2003; Turner et al. 2007; Yang et al. 2005). Furthermore, a decreased risk of lung cancer has been reported with the use of nonsteroidal antiinflammatory drugs, further suggesting a role of inflammation in lung cancer pathogenesis (Khuder et al. 2005; Van Dyke et al. 2008).

Certain hexavalent chromium [Cr(VI)] compounds are occupational and potential environmental human respiratory carcinogens [International Agency for Research on Cancer (IARC) 1990]. Workers in the chromate production industry have an elevated risk of respiratory diseases, including fibrosis, hyperplasia of the bronchial epithelium, lung fibrosarcomas, adenocarcinomas, and squamous cell carcinomas (reviewed by IARC 1990). Outside of commercial use, Cr(VI) is a component of industrial waste, and atmospheric particulate Cr is generated by combustion of fossil fuels, wearing of brake linings, ferrochrome and cement production, ore refining, refractory processing, welding, and incineration of all types [Agency for Toxic Substances and Disease Registry (ATSDR) 2000; Fishbein 1981]. Upon inhalation, Cr particles accumulate at the bifurcations of the bronchi, and the concentration of Cr in these regions of the lung can reach up to 15.8 mg/g of tissue (Ishikawa et al. 1994). Animal studies illustrate that slightly soluble and highly insoluble Cr(VI) particulates such as chromates of zinc, lead, strontium, barium, and sintered calcium consistently induced a tumor response, albeit with variable efficacy (reviewed by IARC 1990).

Aberrant cell survival, proliferation, and tissue remodeling contribute to the etiology of lung cancer (Hanahan and Weinberg 2000). An important survival signaling protein in the lung is the kinase Akt, which is activated by phosphorylation at Ser-473 and Thr-308 (reviewed by Bellacosa et al. 1991). Akt activation initiates a cascade of signaling events that affect neovascularization, proliferation, and inhibition of apoptosis (Altomare and Testa 2005). Increased levels of phosphorylated Akt (phospho-Akt) have been associated with non-small-cell lung cancer, and increased phospho-Akt staining intensity correlates with poor patient prognosis (Altomare and Testa 2005). We have previously shown that a single exposure of particulate Cr(VI) induces toxic mucosal injury and an acute inflammatory response characterized by the up-regulation of interleukin-6 (IL-6) and growth-regulated oncogene-α (Gro-α), phosphorylation of Akt, and infiltration of neutrophils and lymphocytes into the lung (Beaver et al. 2009). Tissue remodeling is critically dependent on matrix metalloproteases (MMPs), which are frequently activated during lung injury, inflammation, and cancer development. Activation of MMPs results in the degradation of collagens in the extracellular matrix (reviewed by Coussens and Werb 2002).

We hypothesize that particulate Cr(VI) will induce an inflammatory microenvironment in the lung that will promote proliferation and selection of growth-altered cells. We investigated the inflammatory processes, tissue injury, and changes in markers associated with dysregulated cell proliferation after repetitive exposure to particulate Cr(VI). We used particulate basic zinc chromate because it is a moderately insoluble form of Cr(VI) that yields a fairly consistent tumor response in mice (Steffee and Baettjer 1965). Furthermore, studies on zinc compounds in cell culture or inhalation exposure demonstrate that zinc has little toxicologic or immunologic consequence in the lung (Wallenborn et al. 2008). Our findings demonstrate that repetitive exposure to Cr(VI) particles induces chronic tissue injury and inflammation that could provide the necessary microenvironment for the development of Cr(VI)-mediated carcinogenesis.

## Materials and Methods

### Chromium preparation and intranasal administration

**Figure 1:**
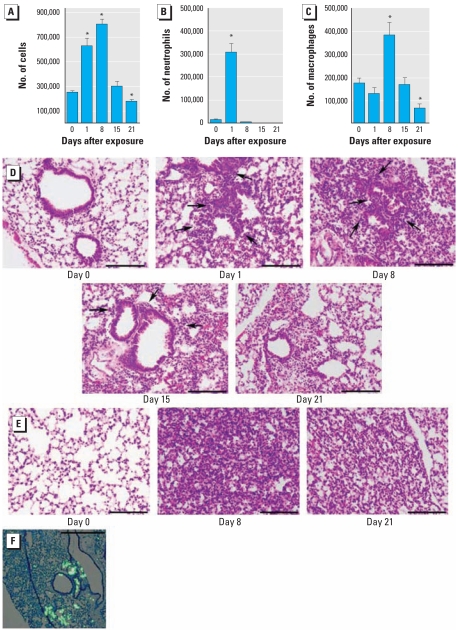
Resolution of inflammation in BAL fluid (*A–C*) and lung sections (*D–E*) at indicated times after a single exposure to particulate Cr(VI) (50 μL of 0.6 mg/mL basic zinc chromate). (*A*) Total number of living cells. (*B* and *C*) Total number of neutrophils (Gr1+; *B*) and macrophages (CD11c+; *C*) determined by FACS analysis. (*D* and *E*) Representative bright-field photomicrographs of H&E-stained lung sections (original magnification, 20×) showing (*D*) airways and (*E*) alveolar regions. Arrows indicate regions of peribronchial inflammation. (*F*) Merged fluorescence (460–500 nm) and bright-field microscopy images of Giemsa-stained lung tissue. Bars = 200 μm. *p < 0.05 compared with control.

**Figure 2:**
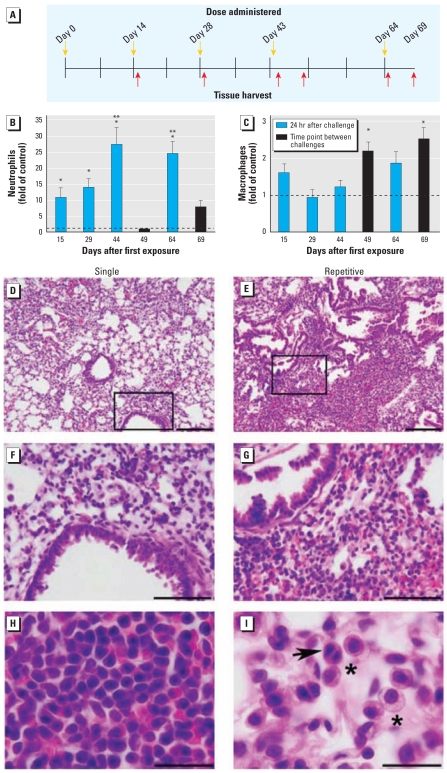
Effects of repetitive Cr(VI) exposure on airway inflammation. (*A*) Regimen of repetitive intranasal exposure of mice to 50 μL saline or 0.6 mg/mL basic zinc chromate suspended in saline. Number of neutrophils (Gr1+; *B*) and macrophages (CD11c+; *C*) in BAL fluid quantified by FACS analysis for 5–11 chromate-treated animals and 6–7 saline-treated animals, expressed as fold of saline control. (*D*–*I*) Bright-field photomicrographs of H&E-stained lung sections obtained from mice 8 days after a single Cr(VI) exposure (*D,F,H*) or 6 days after the fourth exposure (*E,G,I*). Boxed areas in *D* and *E* (10×) are shown at a higher power in *F* and *G* (40×), respectively. (*H*) Lymphocytes and (*I*) macrophages in the lung (100×). Macrophages are indicated by asterisks; the arrow points to a binucleated or dividing macrophage. Bars = 200 μm in *D* and *E*; 100 μm in *F* and *G*; and 30 μm in *H* and *I.* *p < 0.05 compared with same-date saline control. **p < 0.05 compared with Cr(VI)-treated animals at day 15.

Female BALB/cJ mice were obtained from the National Cancer Institute (Frederick, MD) and were 6–8 weeks of age at first Cr(VI) treatment. All experiments were performed in accordance with and under the approval of the George Washington University Institutional Animal Care and Use Committee, and all animals were treated humanely and with regard for alleviation of suffering. We used fluorescent polystyrene particles (4 μm; Phosphorex, Inc., Fall River, MA) as a control to visualize deposition of particles (Figure 1F). Endotoxin-free basic zinc chromate [ZnCrO_4_ 4Zn(OH)_2_] was 4.7 μm in size and had a purity of 99–100% (Rockwood Pigments, Beltsville, MD) (Beaver et al. 2009). Cr(VI) was suspended in sterile 0.9% sodium chloride solution at a concentration of 0.6 mg/mL and prepared as previously described (Beaver et al. 2009). Animals under a light anesthesia (isoflurane) were intranasally exposed to a 50-μL dose of chromate or saline and sacrificed by exposure to carbon dioxide at indicated time points. To determine the duration of injury and inflammation after Cr(VI) exposure, we conducted a set of experiments up to 21 days after a single Cr(VI) treatment. Repetitive Cr(VI) studies were conducted after the described exposure regimen (Figure 2A) for up to 69 days after the initial Cr(VI) exposure. Lungs of animals from both the single and repetitive exposure experiments were further analyzed for inflammation and injury.

### Flow cytometry and cytokine analysis

Mice were euthanized, airway cells were collected from lungs at indicated time points by bronchoalveolar lavage (BAL), and the remaining live cells were quantified and stained for fluorescence-activated cell sorting (FACS) analysis to detect neutrophils (Gr1), eosinophils (FcεRIα), T lymphocytes (CD3, CD4, and CD8), B cells (B220), alveolar macrophages (CD11c), and tissue macrophages (CD11b), as previously described (Beaver et al. 2009). Enzyme-linked immunosorbant assays (ELISAs) were performed on the supernatant of BAL fluid following manufacturer’s instructions to assess the presence of pro-MMP9 and the cytokines IL-6 and tumor necrosis factor-α (TNF-α) (ELISA kits from R&D Systems, Minneapolis, MN).

### Histology

After BAL, whole lungs from each mouse were perfused with 20 mL chilled saline to remove red blood cells. Lungs were then fully expanded with 1 mL periodate-lysine-paraformaldehyde fixative (0.01 M sodium *m*-periodate, 0.075 M lysine, 1% paraformaldehyde) introduced through the trachea, which was tied closed with suture string to maintain the fully expanded state. To obtain complete fixation, tissues were submerged in fixative overnight and transferred into 70% ethanol. Fixed lung tissue was paraffin embedded, sectioned (4 μm), and stained by hematoxylin and eosin (H&E) (HistoServ, Germantown, MD) and Giemsa (Sigma-Aldrich, St. Louis, MO), as previously described (Beaver et al. 2009). Immunohistochemistry with phospho-specific serine-473 Akt antibody (Cell Signaling, Danvers, MA) and Ki-67 antibody (MIB-1 clone; Dako, Carpinteria, CA) was also performed (HistoServ) (Tsurutani et al. 2006). Bright-field photomicrographs were obtained using an Olympus DP70 microscope digital camera (Olympus, Center Valley, PA) with supporting DPController and DPManager software on an Olympus Provis AX70 microscope with the indicated objective magnification.

### Statistical analysis

To determine significant differences among experimental groups, we performed statistical analyses with GraphPad Prism (GraphPad Software Inc., La Jolla, CA). We used two-tailed, unpaired *t*-tests when comparing two experimental groups. One-way analysis of variance and a Tukey or Dunnett’s posttest was performed for multiple sample comparisons. Results are presented as the mean ± SE, and *p* < 0.05 is considered significant.

## Results

### Persistence of inflammation after Cr(VI) exposure

In the present study, we first determined the kinetics of resolution of inflammation after a single Cr(VI) exposure to establish an optimal time course for subsequent exposures. Mice were intranasally exposed to basic zinc chromate, and the number of cells in BAL fluid was evaluated as an initial measure of lung inflammation. We observed a significant increase in the total number of viable cells—approximately 2.5 times the number of cells—in BAL fluid 1 and 8 days after chromate exposure compared with day 0 (Figure 1A). Cell numbers in BAL fluid returned to control levels by 15 days after particulate Cr(VI) treatment and further declined by 21 days after treatment. A significant recruitment of neutrophils into BAL fluid was apparent 1 day after Cr(VI) exposure (Figure 1B), consistent with our previous observations (Beaver et al. 2009). This increase in neutrophils resolved within 7 days and was followed by an influx of macrophages into the BAL fluid that was apparent 8 days after Cr(VI) exposure (Figure 1C). The number of macrophages then returned to a basal level 15 days after treatment and was significantly less than control samples 21 days after Cr(VI) exposure. We observed no T lymphocytes or eosinophils at any time (data not shown).

The persistence of inflammation was also examined in lung tissue. We observed peribronchiolar inflammation 1 day after Cr(VI) exposure, which increased up to 8 days after intranasal treatment (Figure 1D). Peribronchiolar inflammation began to resolve 15 days after Cr(VI) exposure and was no longer apparent 21 days after treatment. Alveolar and interstitial inflammation was detected, consistent with a Cr(VI)-induced pneumonitis (Figure 1E). The alveolar inflammation was most prominent 8 days after Cr(VI) treatment but remained detectable up to 21 days after exposure. Both peribronchiolar and alveolar inflammation were centrally located, whereas near-pleural regions remained uninvolved. Indeed, intranasal delivery of fluorescent beads, similar in size to zinc chromate, resulted in deposition that was primarily localized in foci in and around the respiratory and terminal bronchioles and the proximal alveolar ducts (Figure 1F). Furthermore, most of the particles were centrally located in the lung. Overall, these data illustrate that a single intranasal exposure to particulate Cr(VI) induces a significant inflammatory response in the lung that begins to resolve 15 days after Cr(VI) treatment, although inflammatory cells are still detectable up to 3 weeks after exposure.

### Cr(VI)-induced chronic inflammation

Workers in the chromate industry are repetitively exposed to particulate chromates (IARC 1990). To examine the inflammatory response in the lung after repetitive particulate Cr(VI) exposure, we developed an intranasal regimen of basic zinc chromate or saline treatment (Figure 2A). This dosing regimen was chosen because inflammation began to resolve 2 weeks after a single Cr(VI) exposure (Figure 1). We evaluated the total number of cells in BAL fluid, which was consistently elevated at all time points examined (data not shown). A significant recruitment of neutrophils into lung airways was apparent at all time points that were 24 hr after Cr(VI) exposure (Figure 2B). Although we observed a 10-fold increase in neutrophils compared with saline controls after the first and second Cr(VI) exposures, even greater increases (25-fold) were detected after the third and fourth Cr(VI) exposures. In contrast, no significant increase in neutrophil numbers was detected in the airways of mice sacrificed at time points between Cr(VI) exposures (Figure 2B). Macrophage numbers were significantly elevated (2-fold increase) in the airways between Cr(VI) challenges (Figure 2C). A small number of CD4+ or CD8+ T lymphocytes were occasionally detected in the airways, but these accounted for < 4% of the total immune cells in Cr(VI)-exposed mice (data not shown). No eosinophils (FcεRIα positive) were observed either in the BAL fluid or in the peripheral blood of Cr(VI)-exposed animals (data not shown). Taken together, these data provide evidence of persistent inflammation of airways after repetitive particulate Cr(VI) exposure.

To further understand the extent of inflammation induced by repetitive particulate Cr(VI) exposure, we examined gross changes in injury to airways and infiltration of immune cells. Peribronchiolar, alveolar, and interstitial inflammation was apparent in all Cr(VI)-exposed mice, at all time points analyzed (Figure 2D–G). Peribronchiolar inflammation was predominantly localized around airways 200–400 μm in size. Although marked leukocytic infiltrates were detectable 8 days after the first Cr(VI) exposure, the extent of inflammation was further increased after multiple Cr(VI) treatments (Figure 2D–G). The inflammation remained centrally located in the lung, whereas near-pleural regions remained unaffected. The most abundant leukocyte subset detected in Cr(VI)-exposed lung tissue at all time points examined was lymphoid cells (Figure 2H).

Histologic examination also revealed macrophages in the lung tissue of chromate-exposed animals (Figure 2I). Interestingly, after repetitive Cr(VI) exposure, we occasionally observed macrophages that were either binucleated or at the anaphase stage of mitosis (Figure 2I). Overall, the inflammation detected in Cr(VI)-exposed mice indicates chemical-induced pneumonitis. The histologic observations of inflammation were confirmed by FACS analysis of cell suspensions generated from lung tissue. We observed a significant 3.1-fold increase in the number of immune cells present in lung tissue 24 hr after the fifth Cr(VI) exposure, which consisted of lymphoid cells (92.9% of total leukocytes), macrophages (4.6%), and neutrophils (2.6%). Most lymphoid cells were B cells (B220+), followed by a significant number of helper T cells (CD4+) and few cytotoxic T cells (CD8+) (data not shown). Overall, the pathology illustrates that repetitive intranasal exposure to particulate Cr(VI) induces a chronic inflammatory response in the lung.

### Cr(VI)-induced lung injury

**Figure 3:**
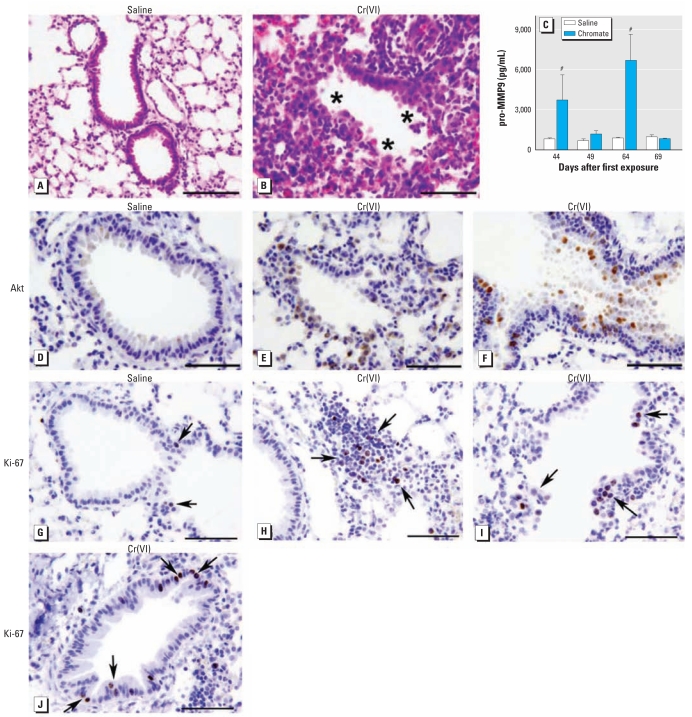
Repetitive Cr(VI) exposure induced airway injury (*A,B*), Akt signaling (*D*–*F*), and epithelial proliferation (*G–J*). Representative bright-field photomicrographs of H&E-stained (*A*,*B*) or immunohistochemically stained (*D–J*) lung sections obtained from mice at 24 hr after the fifth exposure to Cr(VI) (*E*–F and *H–J*) or saline (*A,D,G*). In (*B*) regions of airway with injured epithelium are indicated by asterisks. (*C*) pro-MMP9 levels measured in BAL fluid by ELISA in 5–8 mice. (*D–F*) Tissue sections labeled by phospho-specific serine-473 Akt antibody. (*G*–*J*) Tissue sections labeled by Ki-67 antibody; arrows indicate cells or regions of cells that are positive for Ki-67. Original magnification, 40×; bars = 100 μm. #*p* < 0.0001 compared with the same-day saline control, as determined by *t*-test.

We examined lung tissue of mice to determine the type and extent of lung injury after repetitive particulate Cr(VI) exposure. Proximal and midproximal toxic bronchiolar mucosal injury was present in airways after repetitive Cr(VI) treatment (Figure 3B). This injury was characterized by degenerative changes and sloughing of epithelial cells. The injury was centrally located in the lung, whereas nearby pleural regions remained uninvolved (data not shown). To further characterize the Cr(VI)-induced injury, we measured the levels of pro-MMP9 in BAL fluid. Increases of 4.9- and 7.7-fold were detected in the pro-MMP9 level at 44- and 64-day time points, respectively (Figure 3C). These time points were 24 hr after a Cr(VI) challenge and correlated with the presence of neutrophils in the airways. We observed no difference in pro-MMP9 in the samples collected between Cr(VI) treatment at days 49 and 69 (Figure 3C). No TNF-α or IL-6 was detected in the BAL fluid 24 hr after the fifth Cr(VI) exposure (data not shown).

### Cr(VI)-induced Akt signaling and epithelial proliferation

Mice intranasally exposed to particulate Cr(VI) up-regulated Akt phosphorylation at 1 and 24 hr after treatment (Beaver et al. 2009). We hypothesized that repetitive particulate Cr(VI) exposure could also induce a survival signaling mechanism in airway epithelial cells following Cr(VI) injury. Therefore, we measured phosphorylation of Akt on Ser-473 at 24 hr after the fifth Cr(VI) or saline exposure. A basal level of phospho-Akt was detected in animals exposed to saline alone (Figure 3D). Repetitive Cr(VI) treatment resulted in increased intensity of phospho-Akt staining in epithelial cells lining airways injured by Cr(VI) exposure (Figure 3E). Additionally, phospho-Akt was detected in some inflammatory cells that were present in the Cr(VI)-treated lungs (Figure 3E). Interestingly, phospho-Akt was also detected in epithelial cells lining airways that displayed aberrant growth (Figure 3F).

Given the repetitive injury and presence of survival signaling in cells of particulate Cr(VI)-exposed airways, we postulated that cell proliferation would be induced. Ki-67 is extensively used as a proliferation marker, and a few Ki-67–positive cells were detected in airways of mice exposed to saline alone, representing a basal level of proliferation (Figure 3G). We observed an increase in Ki-67–positive cells in both injured (Figure 3I) and intact (Figure 3J) airways in response to repetitive particulate Cr(VI) exposure. Interestingly, Ki-67–positive lymphoid cells were also detected in the lungs of mice repetitively exposed to particulate Cr(VI) (Figure 3H).

**Figure 4:**
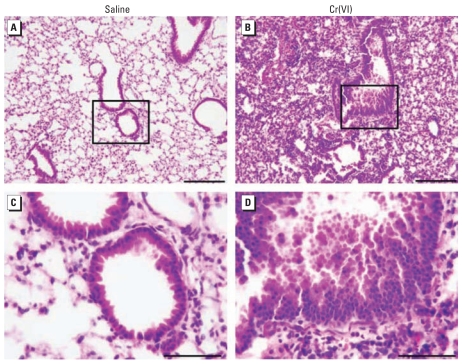
Proliferative response in epithelial cells repetitively exposed to particulate Cr(VI) shown by representative bright-field photomicrographs of H&E-stained lung sections obtained from mice 24 hr after the fifth saline or Cr(VI) exposure. (*A,B*) Original magnification of 10×; bars = 200 μm. (*C,D*) Boxed areas in *A* and *B,* respectively. Original magnification of 40×; bars = 100 μm.

Lungs of Cr(VI)-exposed mice and saline controls were examined for any early signs of uncontrolled cell growth. We detected a proliferative response in epithelial cells lining the airways of mice repetitively exposed to particulate Cr(VI) (Figure 4). In these airways, the epithelial cells no longer formed a monolayer lining the airway, but rather demonstrated cellular stratification. This proliferative response was detected in most Cr(VI)-exposed animals, whereas it was infrequently observed in mice exposed to saline alone.

## Discussion

Chronic inflammation is involved in the pathogenesis of many cancers, including those of the lung (reviewed by Coussens and Werb 2002; Lin and Karin 2007). In the present study, repetitive particulate Cr(VI) exposure induced lung injury as well as a chronic inflammatory response that can be described as repeating waves of infiltrating leukocytes. At 24 hr after each Cr(VI) treatment, the predominant immune cells in the lung airways and tissue were neutrophils and lymphoid cells, respectively. Five days after Cr(VI) exposure, macrophages and lymphoid cells were the dominant infiltrating leukocytes. The inflammation was predominately centrally located in the lung, which was consistent with the localization of similarly sized fluorescent beads in small airways. This type of localization fits well with reports that the highest concentrations of Cr were detected at the bifurcations of the bronchi among chromate workers (Ishikawa et al. 1994).

The presence of neutrophils and macrophages in airways after repetitive Cr(VI) exposure is consistent with welding fume studies in which a significant increase in neutrophils and macrophages was also detected in the lung of exposed rodents (Antonini et al. 2007; Solano-Lopez et al. 2006; Taylor et al. 2003; Zeidler-Erdely et al. 2008). Timing and duration of neutrophil and macrophage infiltrations varied between the present Cr(VI) study and the welding fume studies, potentially due to the additional presence of iron, manganese, and nickel in welding fumes (Antonini et al. 2007; Solano-Lopez et al. 2006; Taylor et al. 2003). Observed increases in neutrophils and no change in macrophages 24 hr after zinc chromate exposure are also consistent with findings in rats exposed to potassium chromate and barium chromate (Cohen et al. 1998). The observed increase in macrophages several days after Cr(VI) exposure is consistent with chronic inhalation of low-dose soluble Cr(VI) observed in rats (Glaser et al. 1985; Steinhoff et al. 1986).

Neutrophils function at the site of injury to initiate the debridement of damaged tissue, phagocytose any pathogens, and amplify the inflammatory response through production of cytokines (Coussens and Werb 2002; Eming et al. 2007). A major function of macrophages is to continue phagocytosis at sites of tissue injury (Freeman et al. 2007). To this end, neutrophils and macrophages release highly active substances, including reactive oxygen species (ROS) and reactive nitrogen species that may promote a microenvironment that directly damages DNA or interferes with the mechanisms of DNA repair (Azad et al. 2008; Federico et al. 2007). In the context of Cr(VI) exposure, these reactive species may further exacerbate DNA damage in surviving and/or proliferating epithelial cells and thus promote initiating events in Cr(VI) carcinogenesis (O’Brien et al. 2003). Macrophages also produce cytokines and growth factors in order to stimulate cell proliferation and angiogenesis (reviewed by Eming et al. 2007).

Neutrophils release proteases in response to injury, including MMPs, to degrade the collagens of the extracellular matrix (Coussens and Werb 2002; Eming et al. 2007). Here we report a correlation between neutrophil presence and pro-MMP9 levels in airways after repetitive particulate Cr(VI) exposure. MMP9 has been shown to play important roles in other lung diseases and is critical in neutrophilic inflammation after ventilator-induced lung injury (Kim et al. 2006). MMPs have been shown to promote inflammation and fibrosis in asbestos-induced lung injury in mice (Tan et al. 2006). It should be noted that expression and activation of MMP9 by macrophages can be induced indirectly, as well as directly, by ROS secreted by inflammatory cells (Svineng et al. 2008). Thus, ROS may participate not only in neoplasia but also in perpetuating an inflammatory microenvironment that actively facilitates tumor progression in the lung. Taken together, we propose that the repeated influx of neutrophils followed by the chronic presence of macrophages leads to tissue that is repetitively injured, thereby promoting a microenvironment that actively facilitates Cr(VI)-induced neoplastic progression in the lung. In support of this, a recent study in mice also suggests that a chronic inflammatory response to welding fumes may enhance tumorigenesis in the lung (Zeidler-Erdely et al. 2008).

Similar to Cr(VI), chronic exposures to asbestos, nickel, and beryllium have been associated with inflammation and increased lung cancer risk (Mossman and Churg 1998; Oller 2002; Salehi et al. 2009). Although some clear differences in these chemicals and the resulting lung pathology exist, it is interesting to note some similarities among particle-mediated lung inflammation (Mossman and Churg 1998). For example, exposure to both Cr(VI) and asbestos particles induced pneumonitis, the recruitment of neutrophils, macrophages, and lymphocytes, and the production of IL-8 in lung airways (Chapman et al. 2003). The detailed mechanisms are not completely understood, but a pattern between chronic lung inflammation and increased cancer risk is clearly emerging in the literature.

A small portion of the lymphoid cells recruited to the lung after Cr(VI) exposure were positive for the proliferation marker Ki-67. Proliferating lymphoid cells have been observed in lung tissue under pathologic conditions including follicular bronchiolitis, lymphoid interstitial pneumonia, chronic hypersensitivity pneumonitis, asthma, and malignant lymphoma (Nicholson et al. 1995; Suda et al. 1999). We also observed binucleated or dividing macrophages, which is consistent with the literature on exposure of the lung to other carcinogens and is indicative of clastogenic/cytogenetic damage to alveolar macrophages (Balansky et al. 1999). Further studies are needed to understand the mechanism that might be driving the Cr(VI)-induced proliferation of leukocytes.

We also observed that repetitive Cr(VI) exposure resulted in enhanced phosphorylation of Akt in epithelial cells of both injured and proliferative airways. These results are consistent with our previous study showing an increase in lung epithelial Akt phosphorylation at 1 and 24 hr after a single Cr(VI) exposure, although the mechanism by which Cr(VI) up-regulates Akt phosphorylation is not clear at this time. It is possible that the Src family of protein tyrosine kinases and/or the regulatory subunit of phosphoinositide-3-kinase, p85, is activated *in vivo*, in a similar manner to their activation by Cr(VI) in cultured lung cells, thus possibly resulting in Akt phosphorylation (O’Hara et al. 2003; and data not shown). Akt activation has been linked to inflammation, neutrophil migration, protection against oxidant, mechanical-induced lung injury, and increased tissue repair (Lai et al. 2007; Li et al. 2007; Ray et al. 2003). Thus, Cr(VI)-induced up-regulation of Akt may promote inflammation, cell survival, and repair of the airways after lung injury. In keeping with this hypothesis, we observed enhanced Ki-67 staining in proliferative epithelial cells, which is consistent with a role for Akt signaling in promoting cell survival in an environment of genotoxic Cr(VI)-induced injury and inflammation.

## Conclusion

The goal of this study was to gain insight into the disease process by which an occupationally and possibly environmentally relevant human carcinogen induces chronic lung damage. This study demonstrates that repetitive exposure to particulate Cr(VI) induces chronic inflammation and injury in the lung. Furthermore, this Cr(VI)-induced injury and inflammation was associated with enhanced survival signaling and epithelial cell proliferation. Taken together, we suggest that these early disease processes promote a microenvironment that may participate in the initiation and promotion of neoplastic cells and contribute over time to Cr(VI) carcinogenesis.
